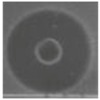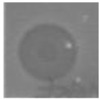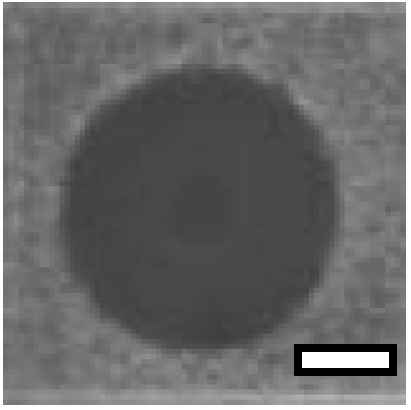# Antimicrobial and Antitumor Activities of Novel Peptides Derived from the Lipopolysaccharide- and β-1,3-Glucan Binding Protein of the Pacific Abalone *Haliotis discus hannai*

**DOI:** 10.3390/md14120227

**Published:** 2016-12-14

**Authors:** Bo-Hye Nam, Ji Young Moon, Eun Hee Park, Hee Jeong Kong, Young-Ok Kim, Dong-Gyun Kim, Woo-Jin Kim, Chul Min An, Jung-Kil Seo

**Affiliations:** 1 Biotechnology Research Division, National Institute of Fisheries Science, 216, Gijanghaean-ro, Gijang-eup, Gijang-gun, Busan 46083, Korea; moonjy@gmail.com (J.Y.M.); jeh8478@naver.com (E.H.P.); heejkong@korea.kr (H.J.K.); yobest12@korea.kr (Y.-O.K.); combikola@korea.kr (D.-G.K.); wj2464@korea.kr (W.-J.K.); ancm@korea.kr (C.M.A.); 2 Department of Food Science and Biotechnology, Kunsan National University, Kunsan 54150, Korea

**Keywords:** antimicrobial peptide, cytotoxic peptide, lipopolysaccharide- and β-1,3-glucan binding protein, *Haliotis discus hannai*

## Abstract

Antimicrobial peptides are a pivotal component of the invertebrate innate immune system. In this study, we identified a lipopolysaccharide- and β-1,3-glucan-binding protein (LGBP) gene from the pacific abalone *Haliotis discus hannai* (HDH), which is involved in the pattern recognition mechanism and plays avital role in the defense mechanism of invertebrates immune system. The HDH-LGBP cDNA consisted of a 1263-bp open reading frame (ORF) encoding a polypeptide of 420 amino acids, with a 20-amino-acid signal sequence. The molecular mass of the protein portion was 45.5 kDa, and the predicted isoelectric point of the mature protein was 4.93. Characteristic potential polysaccharide binding motif, glucanase motif, and β-glucan recognition motif were identified in the LGBP of HDH. We used its polysaccharide-binding motif sequence to design two novel antimicrobial peptide analogs (HDH-LGBP-A1 and HDH-LGBP-A2). By substituting a positively charged amino acid and amidation at the *C*-terminus, the pI and net charge of the HDH-LGBP increased, and the proteins formed an α-helical structure. The HDH-LGBP analogs exhibited antibacterial and antifungal activity, with minimal effective concentrations ranging from 0.008 to 2.2 μg/mL. Additionally, both were toxic against human cervix (HeLa), lung (A549), and colon (HCT 116) carcinoma cell lines but not much on human umbilical vein cell (HUVEC). Fluorescence-activated cell sorter (FACS) analysis showed that HDH-LGBP analogs disturb the cancer cell membrane and cause apoptotic cell death. These results suggest the use of HDH-LGBP analogs as multifunctional drugs.

## 1. Introduction

Invertebrates lack antibodies and an adaptive immune system; instead, they rely on innate immunity to defend themselves against invading pathogens. The innate immune system of marine invertebrates allows them to survive and grow in their microbe-rich benthic environment.

The first stage of the immune response is the recognition of invasive pathogens. Microbial cell-wall components referred to as pathogen-associated molecule patterns (PAMPs), such as LPS, β-1,3-glucan, and peptidoglycans, are recognized by a specific pattern recognition receptors (PRRs) or pattern recognition proteins (PRPs). PRPs bind to PAMPs to form complexes that subsequently activate immune responses such as phagocytosis, nodule formation, encapsulation, activation of proteinase cascades, and synthesis of antimicrobial peptides. To date, various types of invertebrate PRPs, such as peptidoglycan recognition proteins (PGRPs), *C*-type lectins, lipopolysaccharide (LPS)-binding proteins, and β-glucan binding proteins (βGBPs), have been reported.

Lipopolysaccharide- and β-1,3-glucan-binding proteins (LGBPs) consist of two polysaccharide recognition motifs for polysaccharide binding and a β-glucan recognition motif that recognizes bacterial antigens (saccharide moieties) such as LPS, peptidoglycan, and β-1,3-glucan, a major cellular component of yeast and fungi [1]. Several LGBPs have been cloned and characterized in aquatic animals such as crayfish (*Pacifastacus leniusculus*) [2], kuruma shrimp (*Marsupenaeus japonicas*) [3], Chinese shrimp (*Fenneropenaeus chinensis*) [4], Zhikong scallop (*Chlamys farreri*) [5], disk abalone (*Haliotis discus*) [6], and pearl oyster (*Pinctada fucata*)LGBPs [7]. The LGBP of *Pacifastacus leniusculus* was shown to play an important role in prophenoloxidase activation [2].

The LPS-binding or recognition domain has been used to design new antimicrobial peptides (AMPs). For example, the corresponding synthetic LPS-binding domain peptides of anti-LPS factor (ALF) from several crustacean species were shown to exhibit antimicrobial activities [8,9,10,11,12,13]. Lactoferrin is a non-hemic iron-binding glycoprotein with antimicrobial activity via its LPS-binding domain (reviewed by [14]). The recombinant *N*-terminal domain of gram-negative binding protein 3 (GNBP3) binds β-1,3-glucan and shows antimicrobial activity [15]. These studies demonstrated that the antimicrobial properties of the polysaccharide recognition motif can be used to develop novel AMPs. Moreover, recent studies of AMPs have shown that they possess other biological properties, including antiviral and cytotoxic activities [16,17]. In particular, cationic antimicrobial peptides, which are toxic to bacteria but not to normal animal cells, possess a broad spectrum of cytotoxic activity against cancer cells (reviewed by [18]).

In the present study, we identified and designed two novel AMPs based on the polysaccharide-binding domain of the β-1,3-glucan-binding protein of *Haliotis discus hannai*. The antimicrobial activities of these peptides against gram-positive and gram-negative bacteria, as well as yeast, and their cytotoxic activities against three tumor cell lines were examined.

## 2. Results

### 2.1. Identification of the Antimicrobial Peptide and cDNA Sequences

**Figure 1:**
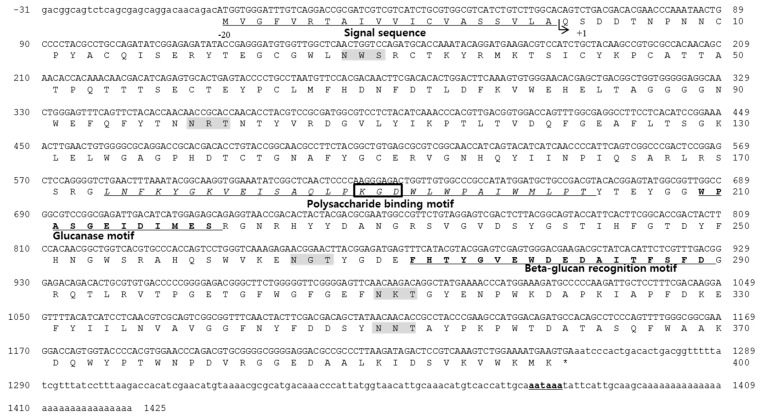
Nucleotide and deduced amino acid sequence of *Haliotis discus hannai* lipopolysaccharide- and β-1,3-glucan binding protein (HDH-LGBP). The sequences are numbered at the right margin of each line. The signal peptide is underlined, and the poly-(A) signal site is bold and underlined. The integrin-binding motif and *N*-glycosylation sites are boxed and highlighted in gray, respectively. The polysaccharide-binding motif is shown in italics and underlined.

By using expressed sequencing tags of *Haliotis discus hannai*, a clone with an incomplete open reading frame (ORF) that showed high similarity to the *Haliotis discus discus* LGBP was isolated. A 632-bp sequence was obtained from clone DGT-151, and the *N*-terminal coding sequence was obtained using the Rapid amplification of cDNA-end (RACE) method and gene-specific primers. The sequence of the 380-bp fragment amplified by 5′-RACE overlapped with an EST sequence to generate the full-length cDNA sequence of the *Haliotis discus hannai* LPS- and β-1,3-glucan binding protein (HDH-LGBP) (Figure 1). The complete sequence of the HDH-LGBP cDNA consisted of a 31-bp 5′-untranslated region (5′-UTR), a 162-bp 3′-UTR with a poly-(A) tail, and a 1263-bp ORF encoding a polypeptide of 420 amino acids with an estimated molecular mass of 47.8 kDa and a theoretical pI of 5.27. The HDH-LGBP gene also encodes a 20-amino-acid putative signal sequence. Therefore, the mature HDH-LGBP consists of 400 amino acid residues with a calculated molecular mass of the protein portion of 45.5 kDa and a predicted pI of 4.93 for the mature protein.

Simple Modular Architecture Research Tool (SMART) analysis revealed that the region corresponding to amino acids 164–301 was similar to that of proteins in the glycoside hydrolase family. Five putative glycosylation sites (Asn–Xaa–Ser/Thr, NXS/T) for *N*-linked carbohydrate chains were identified in the mature protein sequence, at Asn-28, -99, -265, -310, and -350. One of the *N*-linked glycosylation sites was located in proximity to the β-glucan recognition motif, suggesting that glycosylation at this site influences the β-glucan-binding capacity. A short putative cell adhesion site and an integrin binding site, Arg/Lys–Gly–Asp (R/KGD), were also detected in the sequence of the mature protein from Lys-189 to Asp-191. The HDH-LGBP also contained a β-1,3-glucanase site, with Trp-209, Glu-214, Ile-215, and Asp-216 as the active residues (Figure 1).

### 2.2. Peptide Design and Synthesis

**Figure 2:**
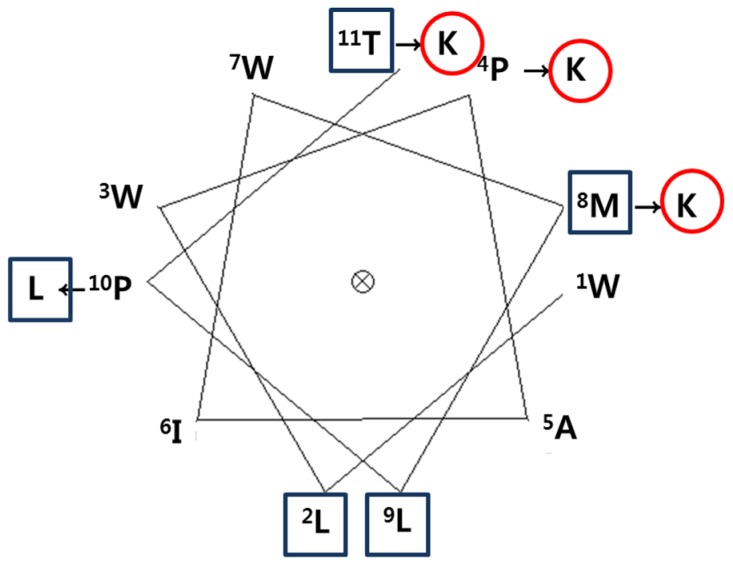
A Schiffer-Edmundson helical wheel representation of HDH-LGBP. The arrows indicate the amino acid residues substituted in the peptide. The hydrophobic and hydrophilic residues are shown in a rectangular box and a circle, respectively.

To develop a novel AMP, we designed a synthetic peptide analog of HDH-LGBP based on the amino acids sequences located in its polysaccharide-binding motif. One native peptide and two analogs were predicted to show antimicrobial activity. The predicted pI, net positive charge, hydrophobicity, and Boman index are listed in Table 1. Peptide activity is influenced by factors such as hydrophobicity, net charge, and the Boman index, which is an estimate of the potential of peptides to bind to other proteins, including receptors. It is defined as the sum of the free energies of the amino acid residue side chains, divided by the total number of amino acid residues. The native parental peptide WLWPAIWKLPT, rich in W and P residues, has an acidic pI value (5.52) and a zero net charge, but its Boman index is low (−2.21). Schiffer-Edmundson helical wheel projections were used to predict the hydrophobic and hydrophilic regions in the secondary structure of the synthetic peptides HDH-LGBP-A1 (WLWKAIWKLLT) and HDH-LGBP-A2 (WLWKAIWKLLK) (Figure 2).

### 2.3. Antimicrobial Activity of HDH-LGBP Analogs

**Figure 3:**
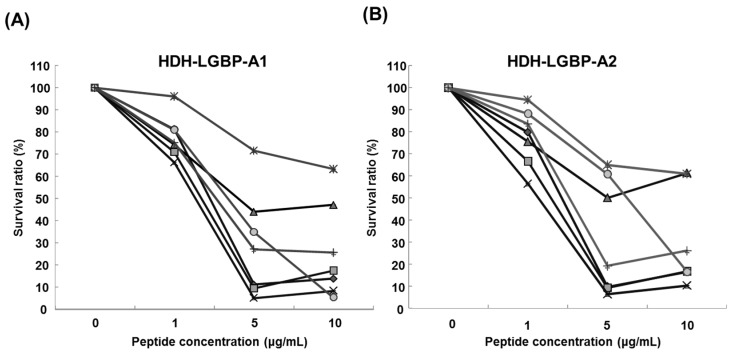
Antimicrobial activity of HDH-LGBP analogs using the broth dilution assay. (**A**) HDH-LGBP-A1; (**B**) HDH-LGBP-A2. Bacterial growth is expressed as a percentage of the maximum optical density (OD) measured in the absence of peptide. Bacterial-killing curve of HDH-LGBP analogs against *B. cereus* (◆), *S. aureus* (■), *S. iniae* (▲), *S. mutans* (**×**), *P. aeruginosa* (*), *V. anguillarum* (●), and *V. harveyi* (**+**). The data were obtained from three independent experiments, each performed in triplicate, and are reported as the mean ± SD.

The antimicrobial activity of the two HDH-LGBP analogs was determined by measuring their minimum effective concentrations (MECs) against gram-positive bacteria, gram-negative bacteria, and the yeast *C. albicans* using URDA (Table 2). The HDH-LGBP analogs showed antimicrobial activity against the gram-positive bacteria *B. cereus*, *S. aureus*, *S. mutans*, and *S. iniae* (MECs 0.008–1.92 μg/mL) and the gram-negative bacterium *P. aeruginosa* (MECs 1.92–2.12 μg/mL), with maximal killing activity at a peptide concentration of 5 μg/mL. By contrast, the antimicrobial activity of the native peptide (HDH-LGBP-N) was low (data not shown). The two analogs also showed potent activity against *C. albicans* (MECs 2.11–2.16 μg/mL). In the liquid culture bacterial growth inhibition test, the curve clearly showed that growth of microorganisms (Gram negative bacteria: *B. cereus*; *S. auresus*; *S. iniae*; *S. mutans*, Gram positive bacteria: *P. aeruginosa*; *V. anguillarum*; *V. harveyi*) was suppressed at 1 μg/mL HDH-LGBP-A1 or -A2, with greater suppression by the two analogs of up to 5 μg/mL (Figure 3). The results demonstrated that the HDH-LGBP analogs have a broad spectrum of antimicrobial activity.

### 2.4. Thermal Stability of HDH-LGBP Analogs

To investigate thermal stability, 5 μg of the synthetic HDH-LGBP peptides/mL were incubated at 100 °C for 10 min and then cooled before they were used in an URDA against gram-positive and gram-negative bacteria and the yeast *C. albicans*. The antimicrobial activity of the peptides was not greatly altered by heat treatment (Table 3), as evidenced by their strong antimicrobial activities against the tested strains (*S. aureus*, *P. aeruginosa*, and *C. albicans*).

### 2.5. Cytotoxicity of HDH-LGBP Analogs

**Figure 4:**
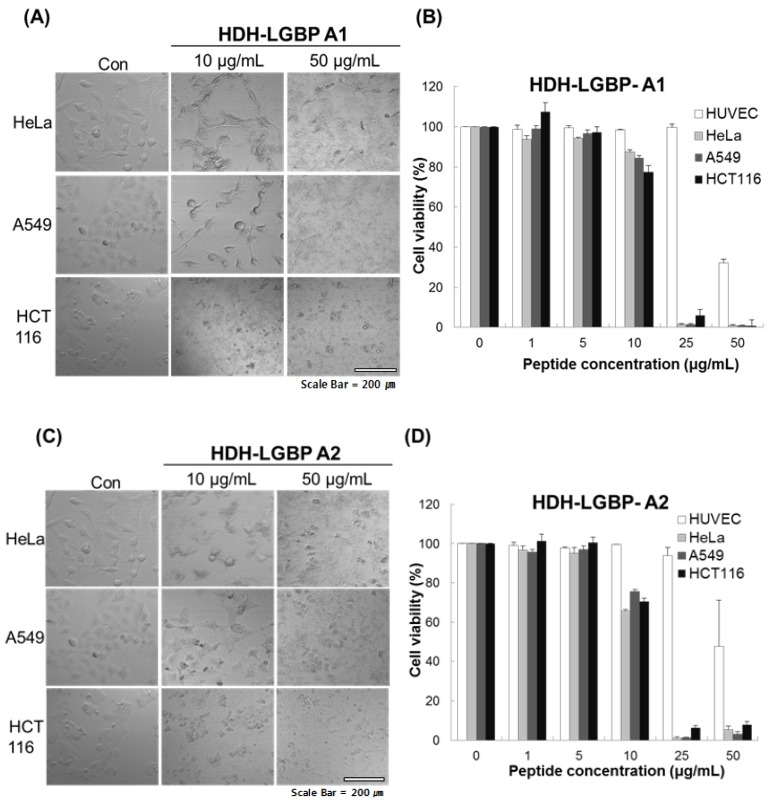
In vitro cytotoxicity of HDH-LGBP analogs. HUVEC, HeLa, A549, and HCT 116 cells were treated with the indicated concentrations of HDH-LGBP-A1 and HDH-LGBP-A2 at 37 °C for 24 h. Cell morphology of HeLa, A549, and HCT116 treated with 10 or 50 μg/mL HDH-LGBP analogs were observed by microscopy (**A** and **C**). Cell viability was measured by an MTS assay after exposure to 0, 1, 5, 10, 25, or 50 μg/mL for 24 h (**B** and **D**). Values represent the mean ± SD (*n* = 3). Scale Bar = 200 μm.

We investigated the cytotoxicity of HDH-LGBP-A1 and HDH-LGBP-A2 on three human cancer cell lines (HeLa, A549, HCT 116 cells) and on a normal cell line, HUVEC, using the MTS assay, which labels live cells based on their mitochondrial dehydrogenase activities, and phase-contrast microscopy. The untreated control cells showed a typical monolayer appearance and had no significant effect on cell viability in the presence of 1–5 μg peptides/mL. However, when the cells were treated for 24 h with 10 μg HDH-LGBP peptides/mL, a decrease in cell number, an increase in the number of rounded cells, and cell shrinkage were observed (Figure 4A,C). In HeLa, A549, and HCT 116 cells treated with 50 μg peptides/mL, cell detachment, swelling, and damage were detected within 5 min (data not shown). This result indicated that higher concentrations (50 μg/mL) of HDH-LGBP-A1 and -A2 directly disrupt the cell membrane. A dose-response experiment showed that treatment of the three cancer cell lines with 1, 5, 10, 25, and 50 μg HDH-LGBP-A1 or -A2/mL for 24 h decreased their viability in a dose-dependent manner (Figure 4B,D). The cytotoxicity of HDH-LGBP-A1 against HeLa cells resulted in 12.4, 98.7, and 99% non-viable cells in cultures exposed to peptide concentrations of 10, 25, and 50 μg/mL, respectively (Figure 4B). The same concentrations also yielded cytotoxic effects in A549 cells (15, 98.5, and 99%) and in HCT 116 cells (22.57, 93.96, and 99%) (Figure 4B). For HDH-LGBP-A2, the corresponding values were 34.4, 99, and 95% in HeLa cells; 24.3, 98.8, and 96.9% in A549 cells; and 29.4, 93.6, and 92% in HCT 116 cells (Figure 4D). At the highest concentration of 50 μg/mL, however, the viability of normal cells was decreased to 32.8% and 47.9% by HDH-LGBP-A1 and HDH-LGBP-A2, respectively (Figure 4D).

### 2.6. Effect of HDH-LGBP on Cancer Cell Membranes

**Figure 5:**
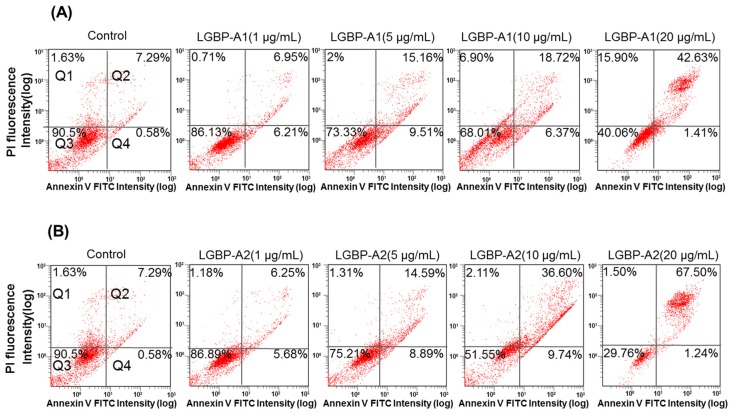
Quantitative analysis of HeLa cells apoptosis and necrosis induced by treatments with abalone HDH-LGBP-A1 (**A**) and HDH-LGBP-A2 (**B**). The cells were incubated with different concentration of 1, 5, 10, and 20 μg HDH-LGBP-A1 and HDH-LGBP-A2/mL for 24 h and then stained with Annexin-V-FITC/PI. Fluorescence intensity was determined using FACS analysis. The upper left part (Q1) represents necrotic cells and the upper right part (Q2) represents secondary necrotic and late apoptotic cells and the lower left part (Q3) represents viable cells and the lower right part (Q4) represents early apoptotic cells.

Cell death induced by AMPs is thought to involve membrane disruption [19]. In this study, the cell-membrane effects of the HDH-LGBP analogs were investigated using Annexin V-FITC/PI staining. Figure 5 shows the dose-dependent decreases in the proportion of viable HeLa cells (quadrant Q3) and the corresponding increases in damaged and dead HeLa cells (quadrants Q2 and Q4). The percentage of viable HeLa cells decreased from 90.5% (control) to 86.13 (1 μg/mL), 73.33 (5 μg/mL), 68.01 (10 μg/mL), and 40.06% (20 μg/mL) after HDH-LGBPA1 treatment; and to 86.89 (1 μg/mL), 75.21 (5 μg/mL), 51.55 (10 μg/mL), and 29.76% (20 μg/mL) after HDH-LGBPA2 treatment. These results showed that HDH-LGBP analogs disrupt membrane integrity (increased PS exposure) and increase membrane permeability (increase cellular uptake of PI), thereby inducing cell death.

## 3. Discussion

AMPs are generally cationic and amphipathic, which enables them to interact with and disrupt lipid membranes. They are also typically very short (5–40 amino acid residues) and contain relatively large (≥30%) proportions of charged (e.g., Lys and Arg) and hydrophobic residues. Some AMPs, such as lactoferricins and indolicidin, are rich in Trp and Arg residues [20]. Unlike currently available conventional antibiotics, which typically interact with a specific target protein, cationic AMPs tend to target the cell membrane of invading microorganisms, leading to cell lysis and death [21]. Thus, AMPs may provide a new class of therapeutic agents whose activities are complementary to those of existing antibiotics. Moreover, bacteria are unlikely to develop resistance to AMPs.

To develop a novel AMP, we designed cationic analogs corresponding to the polysaccharide-binding domain sequence of the abalone β-1,3-glucan-binding protein. The LPS-binding domain is conserved in some PRPs, and is a useful template for designing a mimetic peptide with potential antimicrobial activity. The putative LPS-binding domain of the anti-LPS factor, a small protein with broad-spectrum antimicrobial activities, is pivotal in its antibacterial activity [22]. The synthetic loop of the LPS-binding domain from the ALFs of mud crab [8], shrimp [9,11,12], and Indian mud crab [10] inhibit both gram-negative and gram-positive bacteria, while that from the ALF of black tiger shrimp protects hematopoietic cell cultures from white spot syndrome virus infection [23]. Li et al. [13] had compared antibacterial and antiviral activities of the LPS-binding domain of seven ALF isoforms from the Chinese shrimp and revealed that an identical Lys residue site was specifically conserved in peptide with antimicrobial activity, suggesting that a certain Lys residue is a key residue in antimicrobial activity.

In the present study, to increase the antimicrobial activity of the LGBP derived peptide, we modified the Pro215, Met219, and Pro221 residues of the parent peptide (HDH-LGBP-N) were substituted with Lys, Lys, and Leu to create HDH-LGBP A1; by substituting the Try222 of HDH-LGBP-A1 with Lys, HDH-LGBP-A2 was created (Table 1 and Figure 2). Unlike the parent peptide, the synthetic peptide analogs exhibited inhibitory activities not only against gram-negative and gram-positive bacteria but also against the yeast *C. albicans*. This may have been due to the increased cationicity (net charge) and hydrophobicity of HDH-LGBP-A1 and HDH-LGBP-A2, which facilitated their penetration of the bacterial membrane. The positively charged region of AMPs presumably interacts with the negatively charged bacterial membrane bilayer to form pores via “barrel-stave”, “carpet”, “toroidal-pore”, or “detergent” mechanisms [21,24,25]. Schiffer-Edmandson helical wheel modeling indicated that our LGBP analogs had a hydrophobic area positioned on one side and a positive region on the opposite side (Figure 2). However, the substitution of Lys residue at *C*-terminus of HDH-LGBP-A2 did not increase the antimicrobial activity (Table 2 and Figure 3).

The antimicrobial and cytotoxic activities of AMPs are mediated by targeting the membrane. To determine the effects of HDH-LGBP on mammalian cells, we investigated the toxic effects of HDH-LGBP-A1 and HDH-LGBP-A2 on normal HUVECs and on three cancer cell lines (HeLa, A549, and HCT 116) (Figure 4). The two peptides showed greater cytotoxic than normal-cell toxicity, as determined by comparison of the number of lysed cells. Flow cytometry showed that the two analogs bind to cancer cells and interrupt the cell membrane; thus, the mechanism of the peptides’ cytotoxic effects are similar to that underlying their antimicrobial activities. Like the bacterial cell membrane, the membrane of a cancer cell is rich in negatively charged components such as PS, glycoproteins, and glycosaminoglycans [26]. Accordingly, these negatively charged membrane components favor the binding of positively charged AMPs. Further studies are needed to examine the direct interaction of LGBPs with bacterial and cancer cell membranes and to understand the mechanism underlying the cytotoxic effects of these peptides. In our laboratory, we are currently investigating the cytotoxic mechanisms and activities of HDH-LGBP-A1 and HDH-LGBP-A2.

The therapeutic application of AMPs has been hindered by problems such as toxicity, low stability, and high production costs. Furthermore, the salt sensitivity and thermal stability of AMPs pose major obstacles in their development as novel antibiotics, as many of these peptides lose their antimicrobial activities under physiological salt concentrations and high temperatures [27]. HDH-LGBP-A1 and HDH-LGBP-A2, by contrast, maintained their antimicrobial activities after high-temperature treatment. Therefore, these two analogs may be of value in therapeutic applications.

In conclusion, we successfully designed novel AMPs with high thermal stability and anti-cancer activity using peptide mimetics based on the polysaccharide binding motif of the LGBP of *Haliotis discus hannai*. Synthetic, stable HDH-LGBP-A1 and HDH-LGBP-A2 showed potent antimicrobial activity against bacteria and fungi as well as specific cytotoxicity against cancer, but not normal cells, at concentrations <50 μg/mL. Importantly, because HDH-LGBP-A1 and HDH-LGBP-A2 do not contain non-natural or chemically modified amino acids, they can be produced in a cost-effective manner in biological expression systems. Low in vivo stability, toxicity to mammalian cells, and the high cost of production of most AMPs have prevented their clinical use. However, the absence of these features combined with the antimicrobial and cytotoxic effects of HDH-LGBP-A1 and -A2 demonstrated in this study recommend their further exploration for clinical applications.

## 4. Materials and Methods

### 4.1. Cloning and Sequencing the Full-Length cDNA of Abalone LGBP

cDNA libraries were constructed from seven tissues obtained from three-year-old disk abalones (*Haliotis discus hannai*), and the expressed sequence tags were analyzed as described in a previous study [28]. The sequence of the 632-bp EST clone DGT-151, isolated from the cDNA library prepared from digestive tract tissues, was homologous to the sequences of the LGBPs of other species. To obtain the full-length cDNA of the LGBP gene, digestive tract cDNAs for the 5′- and 3′-random amplification of cDNA ends (RACE) were synthesized using a SMART RACE cDNA amplification kit (BD Bioscience, San Jose, CA, USA) according to the manufacturer’s instructions. Gene-specific primers for 5′- and 3′-RACE were designed based on the partial sequences of the DGT-151 clone (Table 1). The amplified fragments were subcloned into pGEM-T Easy vector (Promega, Madison, WI, USA) and sequenced using an ABI3130 automatic DNA sequencer (Applied Biosystems, Carlsbad, CA, USA). To complete the full-length sequence of LGBP cDNA, the partial sequences of the 5’- and 3’-ends and the partial sequence of DGT-151 were combined and aligned using GENETYX version 8.0 (SDC Software Development, Tokyo, Japan).

### 4.2. Computational Sequence Analysis

The amino acid sequence was deduced from the obtained cDNA, and the molecular mass and isoelectric point were calculated using GENETYX version 8.0 (SDC Software Development, Tokyo, Japan). Sequence similarities with other known sequences were identified using the BLASTP program from the NCBI [29]. The presence of signal peptides was predicted using SignalP 3.0 [30], and domain searches were conducted in the CD-search in NCBI and Pfam sequence search [31].

### 4.3. Structure Prediction

The secondary structure of the peptides was predicted using the GOR method (ExPASy). The theoretical isoelectric point (pI) and net charge were estimated using the ExPASy server [32]. Helical wheel diagrams were produced using EMBOSS Pepwheel (European Bioinformatics Institute, Cambridge, UK) [33]. The Boman index [34] was calculated according to the online Antimicrobial Peptide Database [35].

### 4.4. Peptide Design and Synthesis

A peptide with the amino acid sequence WLWPAIWMLPT, corresponding to the polysaccharide-binding domain of HDH-LGBP and named HDH-LGBP-N, and two modified analogs (HDH-LGBP-A1 and HDH-LGBP-A2) were designed and synthesized commercially by Peptron, Inc. (Daejeon, Korea); the purity grade was >95%. Briefly, the peptide was synthesized using Fmoc solid-phase peptide synthesis (SPPS) with ASP48S (Peptron, Inc., Daejeon, Korea) and purified using reverse-phase high-performance liquid chromatography with a Vydac Everest C18 column (250 mm × 22 mm, 10 μm; Grace, Deerfield, IL, USA). The fractions were eluted with a water-acetonitrile linear gradient (3%–40% (v/v) of acetonitrile) containing 0.1% (v/v) trifluoroacetic acid. The molecular masses of the purified peptides were confirmed using liquid chromatography/mass spectrometry (HP1100 series; Agilent, Santa Clara, CA, USA). All synthetic peptides were dissolved in 0.01% acetic acid to obtain stock solutions of 1000 μg/mL.

### 4.5. Ultrasensitive Radial Diffusion Assay (URDA) for Antimicrobial Potency

The antimicrobial activity of the purified peptide was assessed as described previously [31]. The antimicrobial activities of the synthetic peptides were tested against the gram-positive bacteria, *Bacillus cereus*, *Staphylococcus aureus* RM4220, *Streptococcu siniae* FP5229, and *S. mutans*; the gram-negative bacteria, *Pseudomonas aeruginosa* KCTC2004, *Vibrio anguillarum*, and *Vibrio harveyi* KCCM40866; and the yeast, *Candida albicans* KCTC7965. The bacterial strains were grown in brain-heart infusion medium (BHI; BD Biosciences, San Jose, CA, USA) at the appropriate temperature (25 °C for *P. aeruginosa* and *S. iniae*, and 37 °C for the other strains). The yeast strain *C. albicans* KCTC7965 was grown in yeast medium (YM) at 25 °C. After 16–18 h of incubation, the bacterial and *C. albicans* suspensions were diluted to a McFarland turbidity standard of 0.5 (Vitek Colorimeter #52-1210; Hach, Loveland, CO, USA) corresponding to ~10^8^ CFU/mL for bacteria and ~10^6^ CFU/mL for *C. albicans*. A 500-mL aliquot of the diluted bacterial or *C. albicans* suspension was added to 9.5 mL of underlay gel containing 5 × 10^6^ CFU/mL or 5 × 10^4^ CFU/mL in 10 mM phosphate-buffered saline (PBS; pH 6.6) with 0.03% Tryptic Soy Broth (TSB) or 0.03% Sabouraud Dextrose Broth (SDB) and 1% type I low-EEO agarose. The purified peptide was serially diluted twofold in 5 μL of acidified water (0.01% HAc), and each dilution was added to 2.5-mm-diameter wells made in the 1-mm-thick underlay gels. After a 3 h incubation at either 25 °C (*P. aeruginosa*, *S. iniae*, and *C. albicans*) or 37 °C (the other strains), the bacterial or yeast suspension was overlaid with 10 mL of double-strength overlay gel containing 6% BHI or 6% YM prepared in 10 mM PBS (pH 6.6) and using 1% agarose. The plates were incubated for an additional 18–24 h, after which, the clearing zone diameters were measured. After subtracting the diameter of the well, the clearing zone diameter was expressed in units (0.1 mm = 1 U).

### 4.6. Minimal Effective Concentration of the GBP-Derived Analogs

All tested bacteria and yeast were prepared as described above. The minimal effective concentration (MEC, μg/mL) of the synthetic peptides was calculated as the *x*-intercept of a plot of the above-described units against the log10 of the peptide concentration [36,37]. The antimicrobial assay was performed in triplicate, and the results were averaged.

### 4.7. Effect of Temperature on Antimicrobial Activity

To explore thermal stability, the LGBP analogs were incubated at 100 °C for 10 min, cooled, and then used in the above-described URDA against the bacteria, *B. cereus*, *S. aureus*, *S. iniae*, and *P. aeruginosa*; and the yeast, *C. albicans*.

### 4.8. Cell Culture

Primary umbilical vein endothelial cells (HUVEC; normal human cells), HeLa (human cervical adenocarcinoma), A549 (human lung adenocarcinoma), and HCT 116 (human colorectal carcinoma) cell lines were purchased from the American Type Culture Collection (ATCC; Rockville, MD, USA). HUVEC cells were maintained in vascular cell basal medium (ATCC PCS-100-030) containing Plus One endothelial cell growth factor (ATCC PCS-100-040), and 100 U antibiotics-antimycotics/mL (Life Technologies, Carlsbad, CA, USA) at 37 °C in a 5% CO_2_ incubator (SANYO, Moriguchi, Osaka, Japan). The three cancer cell lines were maintained in DMEM (Welgene, Gyeongsan, Korea) containing 10% fetal bovine serum (Gibco, Grand Island, NY, USA) and 100 U antibiotics–antimycotics/mL (Life Technologies, Carlsbad, CA, USA) at 37 °C in a 5% CO_2_ incubator.

### 4.9. Cell Viability

The cytotoxicity of the AMPs in HUVEC, HeLa, A549, and HCT 116 cells was determined individually using an MTS assay, according to the manufacturer’s instructions of CellTiter 96^®^ Aqueous One Solution Cell Proliferation Assay (Promega, Mannheim, Germany). Briefly, HUVEC, HeLa, A549, and HCT 116 cells (4 × 10^3^ cells/well) were cultured at 37 °C in 96-well plates (Corning, New York, NY, USA) overnight and then incubated for an additional 24 h with 1, 5, 10, 25, or 50 μg/mL of HDH-LGBP-A1 or -A2. Cells in the control group were incubated with 0.01% acetic acid. At the end of the treatment period, 20 μL of a mixture of MTS and the electron-coupling reagent phenazinemethosulfate (Promega, Mannheim, Germany) was added, and the cells were incubated for 4 h at 37 °C. A microtiter plate reader (Perkin Elmer, Waltham, MA, USA) was used to measure the absorbance at 490 nm. The experiment was performed in triplicate and in three independent experiments. The results are expressed as the percentage inhibition of viable cells. Negative control (0.01% acetic acid) values were subtracted from the experimental results.

### 4.10. FITC-Annexin V and Propidium Iodide (PI) Staining

To evaluate the effects of HDH-LGBP on cell membrane integrity and cell-surface phosphatidylserine (PS) exposure, HeLa cells seeded in a 35-mm dish (3.5 × 10^5^ cells/dish) (Corning, New York, NY, USA) and incubated at 37 °C for 24 h were treated with HDH-LGBP-A1 or -A2 at concentrations of 1–50 μg/mL or with 0.01% acetic acid (negative control). After 24 h, the cells were harvested by tryptic digestion, washed with cold PBS, resuspended in binding buffer (0.01 M Hepes/NaOH (pH 7.4), 0.14 M NaCl, 2.5 mM CaCl_2_), and stained according to the manufacturer's instructions with FITC-annexin V and PI (FITC-Annexin V apoptosis detection kit, BD Biosciences). The stained cells were gently mixed and evaluated by flow cytometry (FC500, Beckman Coulter). The results were analyzed using Cell Quest software (BD Biosciences, San Jose, CA, USA). During the early stage of apoptosis, PS shifts from the inner to the outer layer of the plasma membrane. Annexin V, a calcium-dependent, phospholipid-binding protein, binds to PS with high affinity, providing a marker of cell apoptosis. Viable cells with an intact membrane exclude PI, whereas the disrupted membranes of damaged or dead cells are permeable to the dye. The Q1, Q2, Q3, and Q4 gates represented dead cells, the late stage of cell apoptosis, normal cells, and the early stage of cell apoptosis, respectively.

## Figures and Tables

**Table 1:** Sequences and physicochemical properties of the peptides used in this study.

Peptide Name	Sequence	Length	M.W.	p.I.	Hydrophobicity	Hydrophobicmoment	Charge	Boman Index (kcal/mol)	Structure
HDH-LGBP-N	WLWPAIWMLPT-OH	11	1413.7	5.52	−1.62	0.11	0	−2.12	T & R
HDH-LGBP-A1	WLWKAIWKLLT-NH_2_	11	1457.8	10.0	−1.12	0.86	+3	−1.34	H
HDH-LGBP-A2	WLWKAIWKLLK-NH_2_	11	1484.8	10.3	−0.81	1.07	+4	−1.07	H

**Table 2:** Antimicrobial activities of the two HDH-LGBP analogs.

Microbe	Minimal Effective Concentration (μg/mL)		
	Gram	HDH-LGBP-A1	HDH-LGBP-A2
*B. cereus*	+	1.9	1.8
*S. aureus* RM4220	+	1.08	1.37
*S. iniae* FP5229	+	0.57	1.79
*S. mutans*	+	0.008	1.7
*P. aeruginosa* KCTC2004	−	2.12	1.92
*V. anguillarum*	−	>125	>125
*V. harveyi*	−	>125	>125
*C. albicans* KCTC7965	Yeast	2.11	2.16

**Table 3:** Thermal stability of HDH-LGBP analogs against *S. aureus*, *P. aeruginosa*, and *C. albicans*. The upper and lower panels show the radial diffusion assay results of non-heated peptides (N) and of peptides heated for 10 min at 100 °C (H), respectively. Scale bar = 2.3 mm.

Peptide Name	Microbe	*S. aureus*	*P. aeroginosa*	*C. albicans*
HDH-LGBP-A1	N	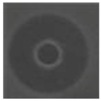	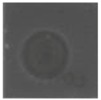	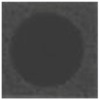
	H	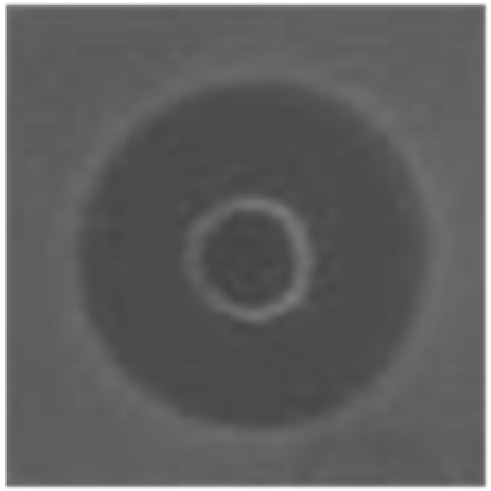	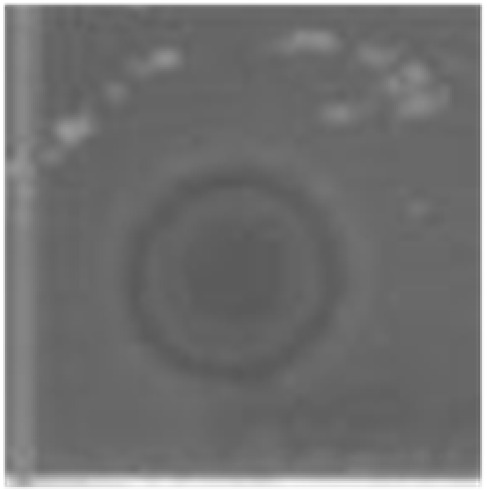	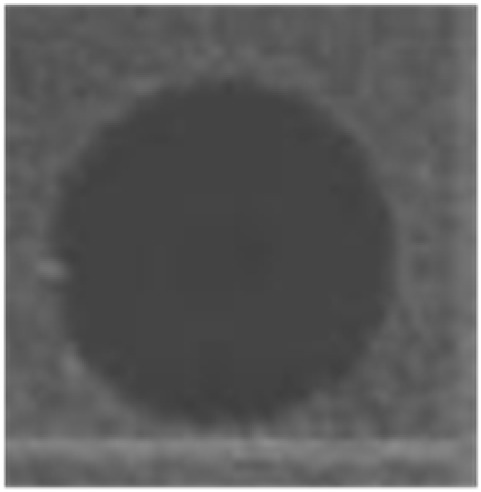
HDH-LGBP-A2	N	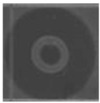	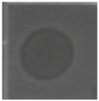	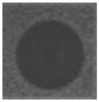
	H